# Supplementary material for: Facile Fabrication of Thin-Bottom Round-Well Plates Using the Deformation of PDMS Molds and Their Application for Single-Cell PCR
Source: Micromachines (Basel). 2020 Jul 31;11(8):748. doi: 10.3390/mi11080748 (PMC7464382; doi:10.3390/mi11080748)
Supplement: Supplementary file 1 [file micromachines-11-00748-s001.pdf]

Supplementary Materials

Facile Fabrication of Thin-Bottom Round-Well Plates Using the Deformation of PDMS Molds and Their Application for Single-Cell PCR

Shinya Yamahira and Yuji Heike

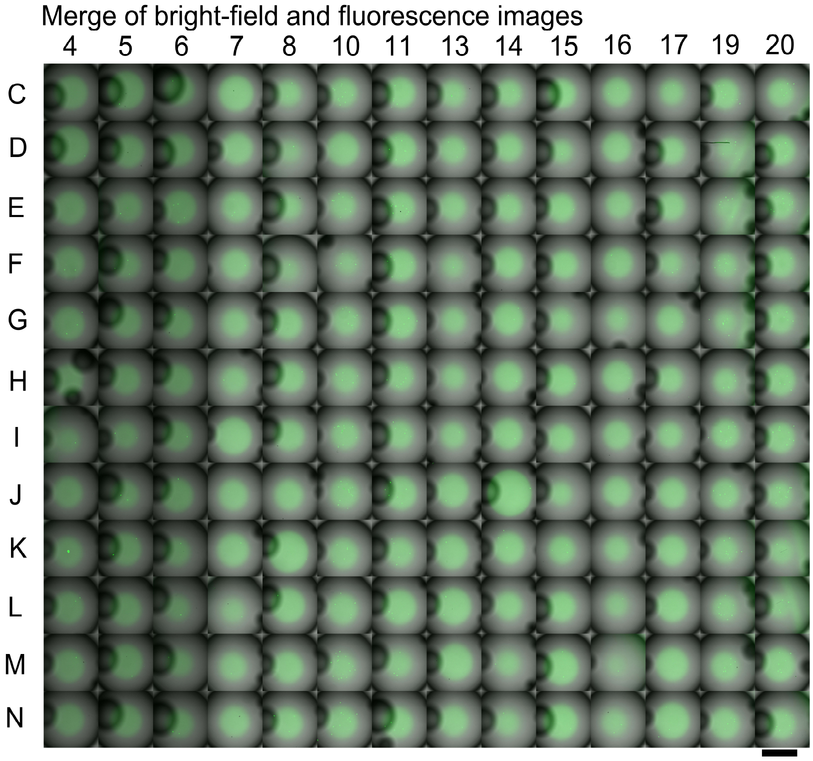

Figure 1. Observation of cells in the PCR mixture on the well bottom (all wells).
